# Non-suicidal Self-Injury in Clinical Practice

**DOI:** 10.3389/fpsyg.2019.00502

**Published:** 2019-03-07

**Authors:** Kirsten Hauber, Albert Boon, Robert Vermeiren

**Affiliations:** ^1^De Jutters, Centre for Youth Mental Healthcare Haaglanden, The Hague, Netherlands; ^2^Curium-LUMC, Oegstgeest, Netherlands; ^3^Lucertis Child and Adolescent Psychiatry, Rotterdam, Netherlands

**Keywords:** NSSI, personality disorder, adolescents, observational descriptive study, MBT

## Abstract

**Background:** Non-suicidal self-injury (NSSI) among adolescents is a major public health concern and a common problem in clinical practice. The aim of this study was to examine different aspects of NSSI in a high-risk adolescent sample in clinical practice in association with personality disorders, symptoms, and coping skills to enhance the understanding of NSSI and improve treatment interventions.

**Methods:** In a sample of 140 adolescent inpatients treated for personality disorders, assessments were performed pre-treatment and post-treatment using a questionnaire on NSSI developed for clinical practice, the Structured Clinical Interview for DSM personality disorders, the Symptom Check List 90, and the Cognitive Emotion Regulation Questionnaire.

**Results:** NSSI was common (66.4%) among the inpatient adolescents. Of those without NSSI behaviour (*n* = 47), 10 (21.3%) started NSSI during treatment. NSSI was related to number of personality disorders and not to one specific. Participants who experienced NSSI (*n* = 93) reported significantly more symptoms and the negative coping strategy self-blame. They scored lower on the positive coping strategies of refocusing and reappraisal.

**Conclusion:** NSSI in adolescent clinical practice is common, not exclusive to borderline personality disorder and could be contagious. Reducing self-blame and enhancing positive refocusing and positive reappraisal seem important treatment targets.

## Introduction

Non-suicidal self-injury (NSSI; e.g., self-inflicted burning, cutting, and punching) among youth is a major public health concern (Glenn et al., 2016). Apparently, it is common but often hidden behaviour, especially among adolescents with psychiatric problems (Madge et al., 2011; Lockwood et al., 2018). Furthermore, it is associated with elevated psychopathology, risk of suicide attempts, and demand for clinical services (Ougrin et al., 2012; Rodav et al., 2014). Knowledge of NSSI in a high-risk adolescent sample can help us better understand this behaviour and optimise prevention and treatment.

Non-suicidal self-injury prevalence is 17.2% among adolescents and 13.4% among young adults (Swannell et al., 2014). The age of onset of NSSI is generally between 12 and 16, and the onset is younger in inpatient adolescents than in outpatient adolescents (Kiekens et al., 2015; Glenn et al., 2016). NSSI is common among adolescents in clinical practice and it is associated with significant functional impairment (Madge et al., 2011). The prevalence rates of NSSI among inpatient adolescents varies from approximately 35 to 80%, depending on numerous methodological variations and different definitions (Madge et al., 2011; Hawton et al., 2012; Zetterqvist, 2015; Koenig et al., 2017). Half of the heterogeneity in these prevalence estimates, can be explained by methodological factors such as measurement errors and differences in assessment and sampling strategies (Swannell et al., 2014).

Also, actual differences in NSSI prevalence between countries may be caused by cultural differences with respect to socio-cultural norms, traditions, as well as substance use policies (Ougrin et al., 2012; Brunner et al., 2013). Cultural differences, likely influence risk factors such as substance use, family integrity and neglect, childhood family adversity, peer rejection, victimisation, and socioeconomic status (Ougrin et al., 2012; Brunner et al., 2013; Giletta et al., 2013; Cassels et al., 2018). Apart from cultural differences, NSSI may also vary as a function of gender, ethnic background, and school-level (Gratz et al., 2012; Hawton et al., 2012). Furthermore, emotional instability in adolescence could partly explain the variability in prevalence rates of NSSI (Kaltiala-Heino and Eronen, 2015).

The distinction between non-suicidal and suicidal self-injury has been a topic of discussion for the last 20 years (Lloyd-Richardson et al., 2009; Zanarini et al., 2013; Grandclerc et al., 2016) due to the fact that most people engaging in NSSI also report suicidal ideation (Klonsky et al., 2013; Whitlock et al., 2013; Glenn et al., 2016). Although most studies consider NSSI an integral feature of borderline personality disorder (BPD), a rapidly growing body of empirical research demonstrates that NSSI co-occurs with a variety of psychiatric disorders, including depression, substance abuse disorders, post-traumatic stress disorder, eating disorders, and other personality disorders (Cawood and Huprich, 2011; Wilkinson, 2013; Gratz et al., 2015; Zetterqvist, 2015). The DSM-5 (American Psychological Association [APA], 2013) defines a NSSI disorder (NSSID) as deliberate, direct, self-inflicted destruction of body tissue without suicidal intent and for purposes not socially sanctioned, engaged on five or more days in the past year. With this definition more covert forms of NSSI behaviours (e.g., self-poisoning) are excluded although several studies showed the existence of forms of NSSI without visible body tissue damage and with psychological damage (Skegg, 2005; Han et al., 2018).

Research on different aspects of NSSI reports that in clinical practice, 87.6% of the adolescents engaging in NSSI have a psychiatric disorder (Nock et al., 2006), and cutting is the most common method (Wilkinson, 2013; Horgan and Martin, 2016). Persons with BPD engaging in NSSI report higher rates of cutting, scratching, head banging, and self-punching than patients without BPD (Turner et al., 2015). In addition, the more methods of NSSI that are used, the higher the risk of suicidal ideation (Wester et al., 2016), accompanied by higher scores for of perceived stress and depressive coping and lower scores for active and optimistic coping (Kiekens et al., 2015). Various functions of NSSI, which are not mutually exclusive, are described in order of prevalence, including affect regulation or reduction of mental pain and transferring mental pain onto the body, self-punishment, influencing other people, anti-dissociation, anti-suicide, and thrill seeking (Klonsky, 2007; Lloyd-Richardson et al., 2009; Glenn and Klonsky, 2010). The contagiousness of NSSI is considered problematic, especially in clinical practice (Bateman and Fonagy, 2006), although to the authors’ knowledge, no research has been conducted on this topic. The Child and Adolescent Self-harm in Europe (CASE) study (Madge et al., 2011) found that higher impulsivity alongside being in connexion with the suicide or self-harm thoughts of others, as well of the occurrence of physical or sexual abuse, worries about sexual orientation and trouble with the police, independently differentiated adolescents who regularly engage in NSSI from single time and non-NSSI adolescents.

Finally, the financial burden of NSSI on society is substantial. In the Netherlands in 2011, the direct medical costs of self-inflicted injury including NSSI was estimated at 60 million euros (National Institute for Health and Environment [RIVM], 2016). Moreover, approximately 157,000 young people between 10 and 24 years old visit emergency departments each year for self-injurious behaviours in the United States, resulting in over 200 million dollars in direct annual medical costs (Glenn et al., 2016). Optimising prevention and treatment programmes can reduce the burden of NSSI on individuals and on society.

In the current prospective cohort study, different aspects of NSSI were inquired pre-treatment and post-treatment in a high-risk adolescent sample with clinically diagnosed personality disorders and comorbidity to enhance the understanding of NSSI. In search of treatment targets, personality disorders, coping skills, and symptoms of distress were examined. Therefore the aims of this study were threefold. First, to investigate the occurrence of NSSI in an inpatient adolescent sample. Second, to examine associations between NSSI and personality disorders, symptoms, and copings skills. Third, to examine contagiousness, frequency, method, and function of NSSI for these groups at pre- and post-treatment. For this purpose the following three groups were compared: a group that performed NSSI in the year preceding treatment, a group that did not perform NSSI in the year preceding treatment and during treatment, and a group that did not perform NSSI in the year preceding treatment but started NSSI during treatment. Based on previous studies, it was assumed, first, that NSSI will be highly common in this inpatient sample; second, that NSSI will be associated with several personality disorders, symptoms and negative copings skills; and third, that NSSI will be contagious, highly frequent, and cutting the most common method and emotion regulation the most common function of NSSI. To enhance the understanding of NSSI in clinical practice, this study examines NSSI forms with and without body tissue damage.

## Materials and Methods

### Participants

The 140 participants were voluntary admissions to a partial residential psychotherapeutic institution for adolescents in the urban area of the Hague in the Netherlands. This facility offers 5 days a week intensive mentalisation based treatment (MBT) (Bateman and Fonagy, 2006; Hauber, 2010) with partial hospitalisation to adolescents with personality disorders between the ages of 16 and 23 years, although by exception a 15-year old adolescent was accepted. During this intensive MBT programme with average duration of 1 year with a maximum of 18 month., personality disorders, insecure attachment and symptoms may diminish (Hauber et al., 2017; Hauber et al., 2018). At the start of the treatment, patients were asked to report NSSI behaviour with the aim of investigating and reducing this behaviour during treatment. The 140 patients in the sample (see Table 1) ranged in age from 15 to 22 years (*M* = 17.91, *SD* = 1.66), and female patients (*M* = 17.84, *SD* = 1.58) were (not significantly) younger than male patients (*M* = 18.24, *SD* = 1.96). At pre-treatment, the borderline (35.7%), avoidant (42.9%), and depressive personality disorder (41.4%) according to the SCID-II were most common and in more than half of the cases in combination with one or more other personality disorders. Most participants also had other clinically diagnosed comorbid non-psychotic disorders. All patients followed the treatment on a voluntary basis. Table 1 presents an overview of study population according to gender, DSM-IV clinical assessed Axis I disorders and Axis II personality disorders assessed using the SCID-II.

**Table 1:** Overview of study population on gender, DSM-IV clinically assessed Axis I disorders and Axis II personality disorders according to the SCID-II (*N* = 140).

	*n*	%
**Gender**		
Female	115	82.1
Male	25	17.8
**Axis I disorders**		
Mood disorders	81	58.0
Anxiety disorders^∗^	43	31.0
Eating disorders	18	13.0
Substance dependence	10	7.0
Dissociative disorders	4	3.0
Obsessive compulsive disorder	3	2.0
Attention deficit hyperactivity disorder	11	8.0
**Axis II disorders**		
No PD	31	22.1
One PD	34	24.3
Two PD’s	43	30.7
Three PD’s	21	15.0
Four PD’s	6	4.3
Five PD’s	4	2.9
Six PD’s	1	0.7
Paranoid PD	20	14.3
Schizoid PD	5	3.6
Anti-social PD	2	1.4
Borderline PD	50	35.7
Narcissistic PD	3	2.1
Avoidant PD	60	42.9
Dependant PD	5	3.6
Obsessive compulsive PD	23	16.4
Depressive PD	58	41.4
Passive aggressive PD	7	5.0


*PD, Personality Disorder. ^∗^including posttraumatic stress disorder*.

### Measures

#### NSSI-Behaviour Questionnaire

The Non-Suicidal Self-Injury Behaviour Questionnaire (NSSI-BQ) N (see Appendix 1) is a self-report questionnaire that was developed for clinical practice with adolescents, consisting of nine items. In 2008, a self-report questionnaire specifically for adolescents on the occurrence, frequency, method, function, readiness to quit, and ways to prevent NSSI was lacking in the authors’ clinical practice. Therefore, the NSSI-BQ was developed by the first two authors. The assumption was that if adolescents were facilitated to be open about their self-injurious behaviour, they would obtain more insight into the underlying causes of their behaviour. In addition, monitoring this behaviour as an integrated part of treatment would help them to regulate their emotions in treatment (Klonsky and Muehlenkamp, 2007) and motivate them to diminish their self-injurious behaviour. A comparison of NSSI information in daily reports with NSSI-BQ scores (see Appendix 2) confirmed that NSSI can be registered using a self-report questionnaire. In two thirds of the cases, there was agreement between the department reports and the patients, and the cases without agreement primarily involved NSSI that was reported by the patient but not recorded by the practitioner.

#### SCL-90

The authorised Dutch version of the Symptom Check List 90 (SCL-90) (Derogatis et al., 1973; Arrindell and Ettema, 2003) is a questionnaire with 90 questions and a five-point rating scale ranging from one (not at all) to five (extreme). This questionnaire assesses general psychological distress and specific primary psychological symptoms of distress from the last week. Outcome scores are divided into nine symptom subscales: anxiety, agoraphobia, depression, somatisation, insufficient thinking and handling, distrust and interpersonal sensitivity, hostility, sleeping disorders, and rest. The total score (range 90–450) is calculated by adding the scores of the subscales. The test-retest reliability was reasonable to good (*k* = 0.62 – 0.91) (Arrindell and Ettema, 2003).

#### CERQ

The Cognitive Emotion Regulation Questionnaire (CERQ) (Garnefski et al., 2002) is a questionnaire of 36 items that can be answered on a five-point Likert scale ranging from one (almost never) to five (almost always). The questions refer to an individual’s thoughts after experiencing threatening or stressful events. The items are proportionally divided into nine scales: self-blame, other blame, rumination, catastrophising, positive refocusing, positive reappraisal, acceptance, putting into perspective, and planning. Previous research on cognitive emotion regulation strategies has shown that all subscales have good internal consistencies ranging from 0.68 to 0.86 (Garnefski et al., 2002).

#### VKP

The Dutch Questionnaire for Personality Characteristics (Vragenlijst voor Kenmerken van de Persoonlijkheid) (VKP) (Duijsens et al., 1996) is a questionnaire of 197 questions with answers of “true” or “false.” The purpose of the VKP is to screen for personality disorders according to the DSM-IV. The VKP is acknowledged for its high sensitivity and low specificity (Duijsens et al., 1996) and therefore is recommended (Dingemans and Sno, 2004) as a screening instrument for the Dutch version of the Structured Clinical Interview for DSM personality disorders (SCID-II) (Spitzer et al., 1990). The outcome of the VKP indicates which SCID-II personality disorder sections should be used. In addition, the test-retest reliability (Cohen’s Kappa) of the VKP on categorical diagnoses was moderate (*k* = 0.40) (Duijsens et al., 1996).

#### SCID-II

The SCID-II (Spitzer et al., 1990) is a structured interview with 134 questions. The purpose of this interview is to establish the ten DSM-IV Axis II personality disorders, and depressive and passive-aggressive personality disorder. The language and diagnostic coverage make the SCID-II most appropriate for use with adults (age 18 or over), though it can be used with younger adolescents with minor modifications (Spitzer et al., 1990). Only the sections which were indicated by the outcome of the VKP were applied in the clinical interview. The SCID-II was administered by trained psychologists. The inter-rater reliability (Cohen’s Kappa) of the SCID-II for categorical diagnoses was reasonable to good (*k* = 0.61 – 1.00) (Seqal et al., 1994) and the test-retest reliability was also reasonable to good (*k* = 0.63) (Weertman et al., 2000).

### Procedure

During an 8-year period (2008–2016), all newly admitted patients were approached to participate in the study. After a verbal description of the treatment protocol to the subjects, written informed consent was obtained according to legislation, the institution’s policy, and Dutch law (European Network of Research Ethics Committees [EUREC], 2017). All patients (*N* = 140) agreed to participate and in concordance with the institutional policy, they participated without receiving incentives or rewards. All procedures in this study were aligned with the 1964 Helsinki declaration and its later amendments or comparable ethical standards. According to the treatment protocol, the patients completed a set of web-based questionnaires in the first and last weeks of treatment.

### Statistical Analysis

All analyses were performed using the Statistical Package for the Social Sciences (SPSS) version 23.0 (International Business Machines [IBM] Corp, 2011). Groups were formed consisting of participants with and without NSSI in the year preceding treatment, and of those who started NSSI during treatment. First the no NSSI, NSSI, and NSSI starters group, and the groups with and without measurements at t-2, were compared based on the number of personality disorders using a χ^2^. A χ^2^ test was then performed to compare the frequency of NSSI between participants diagnosed with BPD and participants with other personality disorders. Second, the NSSI groups were compared based on the level of symptoms (SCL-90) using an ANOVA. A *post hoc* test (Bonferroni) was then used for changes in the level of symptoms. Third, the NSSI groups’ coping skills were compared with an ANOVA, *post hoc* test, and *t*-test. Fourth, the method and function of reported NSSI were compared for the NSSI groups using paired *t*-tests pre and post-treatment. To compare the method of NSSI between participants diagnosed with BPD and participants with other personality disorders, a χ^2^ test was performed. To compare the method of NSSI used, a list of reported NSSI behaviour was also composed (Appendix 3). Because the frequency of NSSI pre and post-treatment was assessed at a nominal level analyses were done using a McNemar test. Finally, a binary logistic regression analysis was performed with NSSI at start of treatment vs. non-NSSI at the start of treatment as dependent variable. The variables that differed significantly (*p* < 0.05) between the two groups were included as independent variables.

## Results

Of the total sample of 140 adolescents, 66.4% (*n* = 93, 87.1% females) confirmed that they had committed NSSI behaviour in the year preceding treatment. From this group, significantly more girls (70.4%) than boys (48.0%) admitted to NSSI behaviour (Pearson χ^2^: 4.635, *df* 1, *p* = 0.031). Data on NSSI behaviour were available for 102 participants post-treatment. Of these 102 patients (92.9% females), 72 (70.6%) reported NSSI at the end of the treatment. Furthermore, a small group of participants who did not report NSSI at pre-treatment, reported NSSI at follow-up (13.9%), resulting in a no NSSI group of *N* = 30, a NSSI group of *N* = 62, and a NSSI starters group of *N* = 10. In the group of which NSSI data were missing at t-2, eight patients reported NSSI at t-1. This non-responders group consisted of significantly more boys than the NSSI group with pre and post-NSSI data did (Pearson χ^2^: 6.695, *df* 1, *p* = 0.010). Furthermore, no significant differences in the variables of NSSI behaviour, SCL-90 scores, CERQ-scores, and SCID-II outcomes at t1 were found between the group of non-responders at t2 and the participants who completed both measurements.

### Comparison of NSSI Groups Based on Personality Disorders

Table 2 presents the number of SCID-II personality disorders in the no NSSI, the NSSI, and the NSSI starters group at pre-treatment.

**Table 2:** Number of SCID-II personality disorders in no NSSI, NSSI, and NSSI starters groups at pre-treatment.

Personality disorder	No. NSSI		NSSI		NSSI Starters		Total	
	
	*n*	%	*n*	%	*n*	%	*n*	%
0	6	27.3	11	15.7	3	30.0	20	19.6
1	9	40.9	13	18.6	5	50.0	27	26.5
>1	7	31.8	46	65.7	2	20.0	55	53.9
Total	22	100.0	70	100	10	100.0	102	100.0


*NSSI, Non-suicidal Self-Injury. Fisher’s Exact Test: 13.626, *p* = 0.005*.

The NSSI group significantly differed from the other groups in the number of personality disorders (Fisher’s Exact Test: 13.626, *p* = 0.005).

### Comparison of NSSI Groups Based on Symptoms

Comparing SCL-90 score at pre-treatment between the NSSI group (*M* = 255.7, *SD* = 44.7) with the no NSSI (*M* = 201.6, *SD* = 58.8) and the NSSI starters group (*M* = 227.7, *SD* = 77.1) revealed significant difference in psychological symptoms of distress (total-score SCL-90) (*f* = 9.54, *p* < 0.001) between the NSSI and the no NSSI group (*post hoc*: *p* = < 0.001). At post-treatment the differences between the NSSI group (*M* = 202.6, *SD* = 62.0), the no NSSI (*M* = 155.6, *SD* = 55.1) and the NSSI starters group (*M* = 173.6, *SD* = 65.5) were also significant (*f* = 4.46, *p* = 0.015). *Post hoc* test showed significant difference (*p* = 0.014) between the NSSI group and the no NSSI group.

### Comparison of NSSI Groups’ Coping Skills

Comparing the three NSSI groups using the CERQ at pre-treatment showed significant differences on three scales of the CERQ: Self-blame [NSSI group (*M* = 14.8, *SD* = 3.7), no NSSI (*M* = 10.8, *SD* = 4.5), NSSI starters (*M* = 12.7, *SD* = 4.2), *F* = 8.72, *p* < 0.001]. Positive refocusing [NSSI group (*M* = 8.7, *SD* = 4.0), no NSSI (*M* = 11.1, *SD* = 4.2), NSSI starters (*M* = 10.4, *SD* = 3.3), *F* = 3.19, *p* = 0.046]. Positive reappraisal [NSSI group (*M* = 8.6, *SD* = 3.2), no NSSI (*M* = 11.8, *SD* = 4.9), NSSI starters (*M* = 12.7, *SD* = 3.5), *F* = 9.28, *p* < 0.001]. *Post hoc* test showed significant differences (*p* = < 0.001) between the NSSI and the no NSSI group on Self-blame and Positive reappraisal.

### Logistic Regression

The characteristics gender, number of personality disorders, total score of SCL-90 and the coping skills self-blame, positive refocusing and positive reappraisal differed significantly between the NSSI and the non-NSSI group and were entered into a logistic regression equation. The logistic regression analysis was performed to test the predictive value of the variables on (the dichotomous dependent variable) NSSI vs. non-NSSI. Only gender, self-blame and positive reappraisal significantly predicted membership of the NSSI-group. The model shows the bivariate odds ratios. Three variables (gender, self-blame and positive reappraisal) significantly predicted membership of the NSSI-group. The model as a whole explained 38 % (Nagelkerke R square) of the variance in NSSI, and correctly identified 80.4% of cases. Table 3 shows the results of this logistic regression analysis.

**Table 3:** Predictors of NSSI.

		95% CI for Odds Ratio		
	**B (SE)**	**Lower**	**Odds Ratio**	**Upper**
Constant	-0.91 (1.32)			
Gender (female)	1.71^∗^ (0.80)	1.16	5.50	26.14
Self-blame	0.19^∗^ (0.07)	1.06	1.20	1.36
Positive reappraisal	-0.21^∗^ (0.07)	0.71	0.81	0.92


*NSSI, Non-suicidal Self-Injury. *R*^*2*^ = 0.38 (Nagelkerke). Model χ^*2*^ (3) = 30.76, ^∗^*p* < 0.05*.

### Frequency of NSSI in the NSSI Group

In the NSSI group, the percentage of participants that reported no NSSI in the last month significantly increased from 23.5% (*n* = 16) at pre-treatment to 63.2% (*n* = 43) at post-treatment (McNemar: *p* < 0.001). No significant difference was found comparing the frequency of self-reported NSSI on NSSI-BQ between patients with BPD, patients with other personality disorders, and patients with no personality disorder according to the SCID-II.

### Method of NSSI in the NSSI Group and NSSI Starters Group

In the NSSI group, scratching and cutting were the methods used most frequently for self-harm at pre-treatment (cutting 69.8%, scratching 66.0%, other method 34.0%, pills 32.1%, drinking 29.2%, head banging 29.2%, and burning 16%). For all the patients who admitted to another method of NSSI than those listed, self-punching was the most frequent (see Appendix 3 for a list of categories of potential NSSI behaviours). The NSSI starters group used methods of NSSI that were not listed more often (e.g., self-punching, hair pulling, bumping, substance misuse, physical neglect, eating problems, and sexual activities). Furthermore, patients with BPD used the methods of scratching (Pearson χ^2^: 5.515, *df* 1, *p* = 0.019), drinking (Pearson χ^2^: 4.824, *df* 1, *p* = 0.028), and pills (Pearson χ^2^: 8.564, *df* 1, *p* = 0.003) significantly more often than patients with other personality disorders.

### Function of NSSI in the NSSI Group

At pre-treatment 80.7% (*N* = 106) of the NSSI group understood (see Appendix 1, question 4) why they performed NSSI behaviour. These participants designated their NSSI behaviour as follows: 64.0% designated it to affect regulation, 22.7% to self-punishment, 0.0% to influencing other people, 18.7% to anti-dissociation, 0.0% to anti-suicide, and 0.0% to thrill seeking. At post-treatment, 88.8% (*N* = 71) of the participants understood why they performed NSSI behaviour. They labelled their NSSI behaviour at t-2 as follows: 71.8% attributed it to affect regulation, 29.6% to self-punishment, 1.4% to influencing other people, 12.7% to anti-dissociation, 1.4% to anti-suicide, and 0.0% to thrill seeking.

## Discussion

The aim of this study was to examine the occurrence, frequency, contagiousness, method, and function of NSSI in a high-risk adolescent sample in clinical practice in association with personality disorders, symptoms of distress, and coping skills. At the start of treatment, in light with our first hypothesis, 12-month NSSI was common (66.4%) among inpatient adolescents as was lifetime NSSI (79.1%). In addition, in line with our second hypothesis, NSSI was related to the number of personality disorders and not to a specific personality disorder. Moreover, the frequency of NSSI was found not to significantly differ between patients with BPD, patients with other personality disorders, and patients with no personify disorder. Patients with NSSI (*n* = 93) disclosed significantly more psychological symptoms of distress at the start of treatment. They also reported using more the negative coping skill self-blame, and less positive refocusing and positive reappraisal as coping skills than the no NSSI group and NSSI starters group. Girls were more than five times more likely to perform NSSI behaviour than boys. Self blame increased the change of NSSI with a third, while positive reappraisal reduced the probability by a fifth. Then, concerning the third hypothesis, with great caution NSSI could be contagious among adolescents in clinical practice, as a small group of patients (*N* = 10) started this behaviour during treatment. However, it is premature to come to conclusions concerning contagiousness of NSSI in clinical practice based on these findings due to this small sample size. Scratching and cutting were the methods used most frequently among participants committing to NSSI behaviour. Other forms of NSSI were mentioned both with and without body tissue damage, although most to a much lesser extent. Affect regulation was mostly communicated as a function of NSSI behaviour. However, replication is necessary to determine the reliability and generalisability of these results due to the small sample size in one facility and the not validated self-report instrument used. The results show that NSSI was not specific to BPD, as it was common among adolescents with BPD and adolescents with other personality disorders, such as the avoidant personality disorder and the depressive personality disorder. However, patients with BPD used the methods of scratching, drinking, and pills significantly more than patients with other personality disorders. Furthermore, NSSI was related to the number of personality disorders that a patient had. A rapidly growing body of empirical research demonstrates that individuals in the general population who engage in repeated NSSI often do not meet the criteria for BPD (Turner et al., 2015) and that NSSI co-occurs with a variety of psychiatric disorders, including depression, substance abuse disorders, post-traumatic stress disorder, eating disorders, and other personality disorders (Cawood and Huprich, 2011; Wilkinson, 2013; Gratz et al., 2015; Zetterqvist, 2015). Therefore, these results could be perceived as preliminary support for a distinct and independent NSSI Disorder (NSSID) classification as suggested in the DSM-5 (American Psychological Association [APA], 2013; Glenn and Klonsky, 2013), although according to the results of this study the NSSID definition excludes a small group of NSSI patients that use NSSI methods without body tissue damage. Many participants (87.7%) that admitted to lifetime NSSI at pre-treatment, met the criteria of NSSID. This new proposed category of NSSI could be helpful to reduce problems from the lack of diagnostic specificity for NSSI; to improve the provision of treatment for adolescents who engage in NSSI; and to enhance research on aetiology, treatment, and outcome. Future studies in patients with NSSI, relating to both Axis I and Axis II diagnoses, may shed more light as to a possible validity of an independent NSSI diagnosis.

In this study substantial overlap between the personality disorders was found, which resembles findings in other, mainly adult studies (Tyrer et al., 2011; Chiesa et al., 2017). Due to this substantial personality disorder symptoms overlap in combination with the overlap with adolescence (e.g., confusion, mood swings, or identity conflicts) and with the co-occurrence of Axis I psychiatric disorders, classifying personality pathology in adolescence correctly is problematic, particularly in severely dysfunctional adolescents. For this reason, the current DSM categorisation by type of personality disorder seems arbitrary in adolescents with co-morbidity.

Notable is that the NSSI group relatively often used the coping skills self blame, acceptance, putting in perspective, and less positive re-interpretation and less blaming others. Especially for adolescent girls, it seems important to change the negative coping strategies such as self-blame as adolescence is the period when NSSI, personality disorders (Tyrer et al., 2015; Amoss et al., 2016) and several major mental health disorders develop (Kessler et al., 2005). Prevention and treatment interventions can target these coping skills to prevent NSSI behaviour.

The findings from this study should be interpreted in relation to limitations. Since the NSSI-BQ is a self-developed, self-report questionnaire for studying adolescents in a clinical psychotherapy facility and not in another target group, the psychometric properties remain unclear. Also, the generalisability of our results is limited due to the use of an inpatient sample of one facility. Another limitation is the amount of dropouts in this study despite attempts to reach and motivate them. Only a part of the adolescents that were included could be followed from the start until the end of treatment. Presumably, there was a lack of motivation to participate in this research project without reward. Furthermore, the small number of the NSSI starters group is a limitation due to which it would be premature to come to conclusions concerning contagiousness of NSSI based on these findings. Finally, not all Axis-II disorders and no Axis-I disorders were examined in this study to avoid overloading patients with assessment instruments.

## Conclusion

In conclusion, this study provided preliminary support that NSSI behaviour could be contagious among adolescents in clinical practice. Important prevention and treatment targets seem to improve positive copings skills, such as positive refocusing and positive reappraisal, and reduce the negative coping skill self blame. Since this negative coping strategy self blame was related to NSSI, it could be a marker bearing clinical relevance.

## Data Availability

The datasets used are available from the corresponding author on reasonable request.

## Ethics Statement

All procedures in this study were in accordance with the 1964 Declaration of Helsinki and its later amendments or comparable ethical standards. Both the legal guardians and the adolescents signed informed consents to participate. The data collection used was part of the treatment protocol. Approval by an Ethics Committee was not required as per the local legislation and institutional requirements.

## Author Contributions

KH performed the data collection and wrote the manuscript. AB contributed to the design of the research project, performed the statistical analyses in the study, and revised the manuscript. RV oversaw the research project and reviewed the manuscript. All authors read and approved the final manuscript.

## Conflict of Interest Statement

The authors declare that the research was conducted in the absence of any commercial or financial relationships that could be construed as a potential conflict of interest.
