# Supplementary material for: Non-suicidal Self-Injury in Clinical Practice
Source: Front Psychol. 2019 Mar 7;10:502. doi: 10.3389/fpsyg.2019.00502 (PMC6424099; doi:10.3389/fpsyg.2019.00502)
Supplement: Supplementary file 1 [file Data_Sheet_1.doc]

Appendix 1

*Non-Suicidal Self-Injury Behaviour Questionnaire*

| Date: ……../……../ 20…. | Name: ………………………………………….. |
| --- | --- |

This questionnaire asks about injuring yourself. Self-injury is intentionally hurt yourself, cause sickness or undertake other actions that are not really good for you. Many young people who have psychiatric problems, struggle to deal with their feelings, uncertainties and problems in a healthy way. Sometimes they exhibit self-injurious behavior. To make life more bearable, they suppress these feelings by inflicting damage to themselves. Some cut themselves more than daily, for example, many others drink on weekends. But there are also many who sleep, overeating or buy many things they do not need

Self-harm is a very intimate topic, something most people do not easily talk about. However, we ask you to fill in this form to get an impression of your potential self-injurious behavior in the past and present. There is no right or wrong answer. Therefore complete this questionnaire as honestly as possible. We understand that the answers are very personal and we will therefore confidentially deal with your information. Without your consent, the results will be discussed with anyone outside the team.

1. Have you ever injured yourself?

O Yes

O No.

2. How long ago did you (approximately) for the last time? (circle the correct answer)

| One day or less | A week | A month | Two months | Half a year | A year | For more than one year |
| --- | --- | --- | --- | --- | --- | --- |

3a. How many times have you done this in the past month? (circle the correct answer)

0 1-5 6-10 11-20 21-30 31-40 41-50 more than 50 times

3b. How many times did you ever do this in a time frame of one month? (circle the correct answer)

Less than once 1-5 6-10 11-20 21-30 31-40 41-50 more than 50 times

4. Do you know why you are doing this or did this?

O No

O Yes, namely ……………………………………………………………………….

………………………………………………………………………………………

Proceed to fill in the back of this list.

5. If you injure yourself, how do you do that? (there is more than one answer possible)

O scratching

O cutting

O burning

O head banging

O drinking

O pills

O otherwise, namely ……………………………………………………………………….

6a. You had the urge to injure yourself the last three months, but you did not do so?

O Yes

O No

If so, how many times did that occur?

0 1-5 6-10 11-20 21-30 31-40 41-50 50 times or more

6b. How you can prevent yourself from self- injure?

………………………………………………………………………………………………………

…………… ………………………………………………………………………………………..

7. How many times have you intended last year to stop with self-injuring?

0 1-5 6-10 11-20 21-30 31-40 41-50 50 times or more

8. How honest have you completed this questionnaire?

O completely honest

O a little honest

O completely not honest

9. Do you have more things you want to share about this?

…………………………………………………………………………………………………..

…………………………………………………………………………………………………..

…………………………………………………………………………………………….........

…………………………………………………………………………………………………..

…………………………………………………………………………………………………..

…………………………………………………………………………………………………..

…………………………………………………………………………………………………..

…………………………………………………………………………………………………..

Thank you for your cooperation
