# Supplementary material for: Non-suicidal Self-Injury in Clinical Practice
Source: Front Psychol. 2019 Mar 7;10:502. doi: 10.3389/fpsyg.2019.00502 (PMC6424099; doi:10.3389/fpsyg.2019.00502)
Supplement: Supplementary file 2 [file Data_Sheet_2.docx]

Appendix 2

Comparing NSSI information in daily reports with scores on NSSI-BQ

The NSSI-BQ was filled out every three months from the start of treatment. The scores of two university students on the scoring list of categories of potential NSSI behaviors (see appendix 3) who independently reviewed all the daily reports of a subsample of 70 patients, were compared. The inter-rater reliability was high (Cohen 's kappa = 0.803; *t* = 69.24; *p* = 0.000). Landis and Koch (Landis & Koch, 1977) consider a value above .80 as ‘almost perfect’. The assumptions of this examination was that when patients reported NSSI on the NSSI-BQ while staff members made no mention of it in the daily reports, this meant that patients reported behavior on the questionnaire that remained hidden or was not reported by the staff members

On self-report (NSSI-BQ), 19 patients reported that they had not engaged in NSSI while 51 patients reported that they did (Table 1). Within the group not having engaged in NSSI (*n* = 19), 7 (36.8%) were reported by staff to have shown NSSI behavior. These behaviors could be interpreted as performed with other, not necessarily injurious intentions (drinking, cannabis use, vomiting, not eating and binge eating). Of the group mentioning NSSI on the NSSI-BQ (*n* = 51), 14 patients (20.0%) had no NSSI events in their file (Table A).

Table A: *Comparison between observed and self-reported NSSI*

| *N* = 70 |  | Patient file | |
| --- | --- | --- | --- |
|  |  | NSSI | No NSSI |
|  |  | *n* (*%*) | *n* *(%)* |
| Self-report (NSSI-BQ) | NSSI | 37 *(52.9%)* | 14 *(20.0%)* |
|  | No NSSI | 7 *(10.0%)* | 12 *(17.1%)* |

NSSI = Non-Suicidal Self-Injury

The agreement between the occurrence of observed and self-reported NSSI was 70%. Of the 14 patients (20%) reporting NSSI that was not observed by the staff members, some behavior was evidently self-injuring (scratching; cutting; head banging; injury caused by knocking against objects; and burning) while some behaviors were less evidently self-injuring (drinking, taking pills, cannabis use; N=XX). The number of different behaviors that were reported according to the NSSI-BQ was higher than the number of different NSSI behaviors in the daily reports. The NSSI-BQ average is 2.56 (*SD* = 1.97, range 0-7) and the daily reports average 1.86 (*SD* = 1.93, range 0-6) (t = 2.123; p < 0.05).
