# Supplementary material for: Non-suicidal Self-Injury in Clinical Practice
Source: Front Psychol. 2019 Mar 7;10:502. doi: 10.3389/fpsyg.2019.00502 (PMC6424099; doi:10.3389/fpsyg.2019.00502)
Supplement: Supplementary file 3 [file Data_Sheet_3.doc]

Appendix 3

List of potential non-suicidal self-injury behaviour

Non-suicidal self-injury through

1: scratching

2: cutting

3: burning

4: head banging

5: drinking

6: taking pills

7: self-punching

8: self-biting

9: hair pulling

10: skin picking

11: pinching

12: breaking bones

13: bumping

14: obstructing wound healing

15: pushing objects/nails into skin

16: substance misuse (alcohol and drugs)

17: medicine misuse (for instance neglect of medication, insulin misuse)

18: physical neglect (for instance sleep too much or too little, move too much or too little)

19: eating problems (for instance vomiting, eating too much or too little or not drinking)

20: performing sexual activities

21: swallowing non edible objects

22: isolating oneself (for instance by gaming and watching series)

23: tattooing oneself

24: removing skin

25: other activities (for instance spending too much, walking or running laps, putting oneself in dangerous situations)
